# Supplementary material for: Meta-analysis of the effectiveness and safety of robotic-assisted versus laparoscopic transabdominal preperitoneal repair for inguinal hernia
Source: PLoS One. 2024 Feb 26;19(2):e0298989. doi: 10.1371/journal.pone.0298989 (PMC10896538; doi:10.1371/journal.pone.0298989)
Supplement: Attachment — Submitted filename: PLOS rTAPP Vs lTAPP.pdf [file pone.0298989.s002.pdf]

PLOS ONE

Meta-analysis of the effectiveness and safety of robotic-assisted versus laparoscopic transabdominal preperitoneal repair for inguinal hernia

--Manuscript Draft--

**Manuscript Number:**	PONE-D-23-32269
**Article Type:**	Research Article
**Full Title:**	Meta-analysis of the effectiveness and safety of robotic-assisted versus laparoscopic transabdominal preperitoneal repair for inguinal hernia
**Short Title:**	Robotic vs. Laparoscopic TAPP Repair: Meta-analysis
**Corresponding Author:**	Jian Wei<br>Zigong Fourth People's Hospital<br>Zigong, Sichuan, CHINA
**Keywords:**	robotic surgery; laparoscopic, transabdominal preperitoneal; inguinal hernia, meta-analysis
**Abstract:**	Background: Inguinal hernia is a common global disease. This study aims to investigate the effectiveness and safety of robot-assisted transabdominal preperitoneal repair (RTAPP) and laparoscopic transabdominal preperitoneal repair (LTAPP) for inguinal hernia.<br>Methods: We conducted a thorough search in Cochrane Library, Embase, and PubMed for relevant clinical studies. After applying inclusion and exclusion criteria, the quality of selected studies was assessed using the Jadad scale for randomized controlled studies and the Newcastle-Ottawa scale for observational studies. Meta-analysis was performed using RevMan 5.3 software.<br>Results: A total of ten studies were included, comprising two randomized controlled studies and eight non-randomized controlled studies. Meta-analysis results revealed no statistically significant differences between the RTAPP group and the LTAPP group regarding hospital stay [MD=0.21, 95% CI (-0.09, 0.51), P=0.17], incidence of seroma [OR=0.85, 95% CI(0.45, 1.59), P=0.61], overall complication rate [OR=1.22, 95% CI(0.68, 2.18), P=0.51], readmission rate [OR=1.31, 95% CI(0.23, 7.47), P=0.76], and recurrence rate [OR=0.82, 95% CI(0.22, 3.07), P=0.77]. However, the RTAPP group had longer operation time compared to the LTAPP group [MD=14.02, 95% CI (6.65, 21.39), P=0.0002], and the cost of the RTAPP procedure was higher than that of the LTAPP procedure [MD=4.17, 95% CI (2.59, 5.76), P<0.00001].<br>Conclusion: RTAPP for inguinal hernia is a safe and feasible approach, however, it is associated with increased operation time and treatment costs.
**Order of Authors:**	Hui Dong
	Li Li
	Yue-Juan Li
	Deng-Chao Wang
	Jian Wei
**Additional Information:**	
**Question**	**Response**
**Financial Disclosure**<br><br>Enter a financial disclosure statement that describes the sources of funding for the work included in this submission. Review the submission guidelines for detailed requirements. View published research articles from *PLOS ONE* for specific examples.	The author(s) received no specific funding for this work.

Powered by Editorial Manager® and ProduXion Manager® from Aries Systems Corporation

This statement is required for submission and **will appear in the published article** if the submission is accepted. Please make sure it is accurate.<br><br>**Funded studies**<br>Enter a statement with the following details:<br>• Initials of the authors who received each award<br>• Grant numbers awarded to each author<br>• The full name of each funder<br>• URL of each funder website<br>• Did the sponsors or funders play any role in the study design, data collection and analysis, decision to publish, or preparation of the manuscript?<br><br>Did you receive funding for this work?	
**Competing Interests**<br><br>Use the instructions below to enter a competing interest statement for this submission. On behalf of all authors, disclose any competing interests that could be perceived to bias this work—acknowledging all financial support and any other relevant financial or non-financial competing interests.<br><br>This statement is required for submission and **will appear in the published article** if the submission is accepted. Please make sure it is accurate and that any funding sources listed in your Funding Information later in the submission form are also declared in your Financial Disclosure statement.<br><br>View published research articles from *PLOS ONE* for specific examples.	The authors have declared that no competing interests exist.

Powered by Editorial Manager® and ProduXion Manager® from Aries Systems Corporation

**NO authors have competing interests**<br><br>Enter: *The authors have declared that no competing interests exist.*<br><br>**Authors with competing interests**<br><br>Enter competing interest details beginning with this statement:<br><br>*I have read the journal's policy and the authors of this manuscript have the following competing interests: [insert competing interests here]*<br><br>* typeset	
**Ethics Statement**<br><br>Enter an ethics statement for this submission. This statement is required if the study involved:<br><br>• Human participants<br>• Human specimens or tissue<br>• Vertebrate animals or cephalopods<br>• Vertebrate embryos or tissues<br>• Field research<br><br>Write "N/A" if the submission does not require an ethics statement.<br><br>General guidance is provided below. Consult the submission guidelines for detailed instructions. **Make sure that all information entered here is included in the Methods section of the manuscript.**	N/A

Powered by Editorial Manager® and ProduXion Manager® from Aries Systems Corporation

**Format for specific study types**<br><br>**Human Subject Research (involving human participants and/or tissue)**<br>• Give the name of the institutional review board or ethics committee that approved the study<br>• Include the approval number and/or a statement indicating approval of this research<br>• Indicate the form of consent obtained (written/oral) or the reason that consent was not obtained (e.g. the data were analyzed anonymously)<br><br>**Animal Research (involving vertebrate animals, embryos or tissues)**<br>• Provide the name of the Institutional Animal Care and Use Committee (IACUC) or other relevant ethics board that reviewed the study protocol, and indicate whether they approved this research or granted a formal waiver of ethical approval<br>• Include an approval number if one was obtained<br>• If the study involved *non-human primates*, add *additional details* about animal welfare and steps taken to ameliorate suffering<br>• If anesthesia, euthanasia, or any kind of animal sacrifice is part of the study, include briefly which substances and/or methods were applied<br><br>**Field Research**<br><br>Include the following details if this study involves the collection of plant, animal, or other materials from a natural setting:<br>• Field permit number<br>• Name of the institution or relevant body that granted permission	
**Data Availability**<br><br>Authors are required to make all data underlying the findings described fully available, without restriction, and from the time of publication. PLOS allows rare exceptions to address legal and ethical concerns. See the PLOS Data Policy and FAQ for detailed information.	Yes - all data are fully available without restriction

A Data Availability Statement describing where the data can be found is required at submission. Your answers to this question constitute the Data Availability Statement and **will be published in the article**, if accepted.<br><br>**Important:** Stating 'data available on request from the author' is not sufficient. If your data are only available upon request, select 'No' for the first question and explain your exceptional situation in the text box.<br><br>Do the authors confirm that all data underlying the findings described in their manuscript are fully available without restriction?	
**Describe where the data may be found in full sentences. If you are copying our sample text, replace any instances of XXX with the appropriate details.**<br><br>• If the data are **held or will be held in a public repository**, include URLs, accession numbers or DOIs. If this information will only be available after acceptance, indicate this by ticking the box below. For example: *All XXX files are available from the XXX database (accession number(s) XXX, XXX.*).<br>• If the data are all contained **within the manuscript and/or Supporting Information files**, enter the following: *All relevant data are within the manuscript and its Supporting Information files.*<br>• If neither of these applies but you are able to provide **details of access elsewhere**, with or without limitations, please do so. For example:<br><br>*Data cannot be shared publicly because of [XXX]. Data are available from the XXX Institutional Data Access / Ethics Committee (contact via XXX) for researchers who meet the criteria for access to confidential data.*<br><br>*The data underlying the results presented in the study are available from (include the name of the third party*	All relevant data are within the manuscript and its Supporting Information files.

and contact information or URL).<br>• This text is appropriate if the data are owned by a third party and authors do not have permission to share the data.<br><br>* typeset	
Additional data availability information:	

Powered by Editorial Manager® and ProduXion Manager® from Aries Systems Corporation

Manuscript
Click here to access/download;Manuscript;Manuscript.docx

Meta-analysis of the effectiveness and safety of robotic-assisted versus laparoscopic transabdominal preperitoneal repair for inguinal hernia

Short Title: Robotic vs. Laparoscopic TAPP Repair: Meta-analysis

Hui Dong[a], Li Li[b], Yue-Juan Li[a], Deng-Chao Wang[a], Jian Wei[a]*

[a]Department of General Surgery, Zigong Fourth People's Hospital, Zigong, 643000, Sichuan, China

[b]Department of Pediatric Surgery, Zigong First People's Hospital, Zigong, 643000, Sichuan, China

***Corresponding author**

Jian Wei

Address: Department of General Surgery, Zigong Fourth People's Hospital, 19 Tanmulin Road, Zigong, Sichuan 643000, P.R. China

Tel: +8613568327242

Fax: +8608132302124

E-mail: jianweipuwaike@163.com

Abstract

Background: Inguinal hernia is a common global disease. This study aims to investigate the effectiveness and safety of robot-assisted transabdominal preperitoneal repair (RTAPP) and laparoscopic transabdominal preperitoneal repair (LTAPP) for inguinal hernia.

Methods: We conducted a thorough search in Cochrane Library, Embase, and PubMed for relevant clinical studies. After applying inclusion and exclusion criteria, the quality of selected studies was assessed using the Jadad scale for randomized controlled studies and the Newcastle-Ottawa scale for observational studies. Meta-analysis was performed using RevMan 5.3 software.

Results: A total of ten studies were included, comprising two randomized controlled studies and eight non-randomized controlled studies. Meta-analysis results revealed no statistically significant differences between the RTAPP group and the LTAPP group regarding hospital stay [MD=0.21, 95% CI (-0.09, 0.51), P=0.17], incidence of seroma [OR=0.85, 95% CI(0.45, 1.59), P=0.61], overall complication rate [OR=1.22, 95% CI(0.68, 2.18), P=0.51], readmission rate [OR=1.31, 95% CI(0.23, 7.47), P=0.76], and recurrence rate [OR=0.82, 95% CI(0.22, 3.07), P=0.77]. However, the RTAPP group had longer operation time compared to the LTAPP group [MD=14.02, 95% CI (6.65, 21.39), P=0.0002], and the cost of the RTAPP procedure was higher than that of the LTAPP procedure [MD=4.17, 95% CI (2.59, 5.76), P<0.00001].

Conclusion: RTAPP for inguinal hernia is a safe and feasible approach, however, it is associated with increased operation time and treatment costs.

Key words：robotic surgery; laparoscopic, transabdominal preperitoneal; inguinal hernia, meta-analysis

Introduction

Inguinal hernia is a common global disease, and with the aging population, its incidence continues to rise [1]. The most frequently employed techniques for laparoscopic inguinal hernia surgery are transabdominal preperitoneal repair (TAPP) and totally extraperitoneal repair (TEP) [2]. The emergence of robotic surgical systems has led to significant advancements and groundbreaking changes in various medical disciplines, including general surgery, hepatobiliary surgery, urology, obstetrics and gynecology, cardiovascular surgery, thoracic surgery, and pediatric surgery. These systems have played a pivotal role in promoting the development of minimally invasive surgery and have ushered in a revolutionary era [3,4]. The cutting-edge da Vinci robotic surgical system has gradually gained traction in some countries and regions and is redefining minimally invasive surgery as the third generation of surgical technology, following open surgery and laparoscopy [5,6]. With its unique features, including 3D imaging, a mechanical arm with 7 degrees of freedom, tremor filtration, and more ergonomic design, da Vinci surgery has elevated surgical precision to unprecedented heights and ushered minimally invasive surgery into a new era [7,8]. Currently, it has been successfully applied in abdominal wall incisional hernia repair, paraesophageal hernia repair, and inguinal hernia repair,

demonstrating its technical advantages [9-11]. While several randomized controlled studies and non-randomized controlled studies have compared RTAPP with LTAPP in the treatment of inguinal hernia [12-21], there is limited data from single-center studies, and clinical reports yield inconsistent results. This study employs a meta-analysis approach to include clinical research on RTAPP and LTAPP for inguinal hernia, comparing their effectiveness and safety. It evaluates the utility of RTAPP in the treatment of inguinal hernia and aims to provide evidence-based medicine for the clinical management of inguinal hernia using robotic technology.

Methods

Our systematic review adheres to the Preferred Reporting Items for Systematic Reviews and Meta-Analyses for Protocols guidelines[22], and it is registered under the number INPLASY202390048.

Inclusion and exclusion criteria

Inclusion Criteria

(1) Study Subjects: Individuals diagnosed with inguinal hernia through preoperative physical examination and ultrasound, aged over 18 years, of any gender. (2) Intervention: Either robotic-assisted transabdominal preperitoneal tension-free repair or laparoscopic transabdominal preperitoneal tension-free repair, with no restriction on the type of mesh used during surgery. (3) Study Type: Randomized controlled trials, non-randomized controlled studies (retrospective or case-control studies), limited to publications in English. (4) Outcome Measures: Operation time, hospital stay, cost, incidence of seroma, overall complication rate, readmission rate, recurrence rate.

Exclusion Criteria

(1) Non-comparative studies. (2) Case reports, abstracts, conference reports, reviews. (3) Studies where surgical procedures did not involve either robotic-assisted transabdominal preperitoneal tension-free repair or laparoscopic transabdominal preperitoneal tension-free repair. (4) Studies from which outcome measures could not be extracted. (5) Literature inaccessible in full text.

Retrieval Strategy

A comprehensive computer-based retrieval was conducted on The Cochrane Library, Embase database, and PubMed database. The retrieval period for all databases extended from their inception to April 7, 2023. The search terms used in the databases were: inguinal hernia, groin hernia, hernioplasty, transabdominal preperitoneal (TAPP), robot, robotic. Additionally, in order to obtain further study on this topic, references from the included literature were also reviewed to determine if they met the inclusion

criteria.

Literature Screening and Data Extraction

Literature retrieval and data extraction were carried out independently by two authors. Any disagreements were resolved through discussion or by seeking assistance from a third researcher. The extracted data included: (1) General information: first author, publication year, country, sample size, gender, age, patch type, follow-up duration; (2) Outcome measures: operation time, hospital stay, cost, incidence of seroma, overall complication rate, readmission rate, recurrence rate.

Quality assessment

Two authors independently conducted quality assessments of the included studies, with cross-verification. In cases of disagreement during the evaluation process, disagreements were resolved through discussion or adjudicated by a third author. The quality of included randomized controlled studies was assessed using the modified Jadad scale, which includes four criteria: (1) random sequence generation, (2) allocation concealment, (3) blinding, and (4) withdraws and dropouts. The total score is 7 points, with scores of ≤3 considered low-quality literature and scores of 4-7 considered high-quality literature [23]. Non-randomized controlled studies were assessed for quality using the NOS (Newcastle-Ottawa Scale), with scoring criteria including (1) selection, (2) comparability, and (3) exposure. Scores of 7-9 indicate high-quality studies, scores of 4-6 indicate medium-quality studies, and scores of 1-3 indicate low-quality studies [24].

Statistical analysis

Data from the included literature were combined and analyzed using RevMan 5.3 software. For continuous variables and binary variables in the studies, mean differences (MD) and odds ratios (OR) were calculated as effect measures along with their corresponding 95% confidence intervals (CI). Heterogeneity among the included studies was assessed using the chi-squared ($\chi 2$) test, and the magnitude of heterogeneity was quantified using the I2 statistic. If there was no significant heterogeneity among the studies (I2 ≤ 50%, P ≥ 0.10), a fixed-effects model was used for analysis. If heterogeneity was present (I2 > 50%, P < 0.10), a random-effects model was employed for analysis. For indicators with more than 10 included studies, the potential publication bias was assessed using a funnel plot of the main results. If the plot showed good symmetry, it indicated no significant publication bias [25]. The significance level was set at $\alpha = 0.05$.

Results

Literature Search Results

Initially, a total of 1,033 articles were retrieved from various databases, and an additional 4 articles were identified through manual searches. After reviewing titles and abstracts, 165 duplicate articles were excluded, along with 803 articles unrelated to the research objectives, 36 articles comprising empirical summaries, reviews, and case reports, and 7 articles pertaining to pediatric inguinal hernias. The remaining 19 articles underwent full-text screening, and 4 articles lacked relevant outcome measures, while 5 articles were excluded due to the absence of control groups. Following the sequential screening process described above, a total of 10 articles [10-29] were ultimately included. For a detailed overview of the selection process as shown in Figure 1. The basic characteristics of the included literature are provided in Table 1.

Results of literature quality assessment

Among the included studies, 2 articles [16,21] were randomized controlled trials (RCTs) with a score of 4, indicating high quality. Eight articles [12-15,17-20] were non-randomized controlled studies, and all of them received high-quality ratings based on the Newcastle-Ottawa Scale (NOS). Among these, 2 articles [14,17] scored 8 points, while the remaining 6 articles scored 7 points [12,13,15,18-20]. The detailed scoring results are presented in Table 1.

Meta analysis results

Operation time

Six studies [12,13,15,19-21] reported operation time. There was significant statistical heterogeneity among the studies ($P < 0.00001$, $I2 = 98\%$). A random-effects model was used to combine the effect sizes for the Meta-analysis, which indicated that the RTAPP group had a longer operation time compared to the LTAPP group [MD = 14.02, 95% CI (6.65, 21.39), P = 0.0002], and this difference was statistically significant. Sensitivity analysis, conducted by sequentially excluding individual included studies, showed that the direction of the combined effect value remained unchanged after each exclusion, suggesting the overall stability of the study results, as shown in Figure 2.

Hospital stay

Three studies [12,13,20] reported hospital stay. There was statistical heterogeneity among the studies ($P = 0.05$, $I2 = 67\%$). A random-effects model was used to combine the effect sizes for the Meta-analysis, which indicated that there was no statistically significant difference in hospital stay between the RTAPP group and the LTAPP group [MD = 0.21, 95% CI (-0.09, 0.51), P = 0.17]. Sensitivity analysis, performed by sequentially excluding individual included studies, demonstrated that the direction of the combined effect value remained unchanged after each exclusion, suggesting the overall stability of the study results, as shown in Figure 3.

cost

Three studies [12,13,21] reported on cost. There was significant statistical heterogeneity among the studies ($P < 0.00001$, I2 = 100%). A random-effects model was used to combine the effect sizes for the Meta-analysis, which indicated that the cost in the RTAPP group was higher than that in the LTAPP group [MD = 4.17, 95% CI (2.59, 5.76), $P < 0.00001$], and this difference was statistically significant. Sensitivity analysis was conducted, and when the study by Choi 2023[13] or Prabhu 2020[21] was excluded, the results showed no statistically significant difference in cost between the two groups. Therefore, it suggests that the stability of the results is relatively low, and it is recommended that future researchers conduct more studies on this aspect, as shown in Figure 4.

Incidence of seroma

Four studies[13,17,19,20]reported the incidence of seroma. The incidence of seroma in the RTAPP group was 19/219 (8.7%), while in the LTAPP group, it was 26/273 (9.5%). There was no statistical heterogeneity among the studies (P = 0.38, I2 = 2%). A fixed-effects model was used to combine the effect sizes for the Meta-analysis, which indicated that there was no statistically significant difference in the incidence of seroma between the RTAPP group and the LTAPP group [OR = 0.85, 95% CI (0.45, 1.59), P = 0.61], as shown in Figure 5.

Overall Complication Rate

Four studies [14,17,18,20] reported the overall complication rate. The overall complication rate in the RTAPP group was 28/522 (5.4%), while in the LTAPP group, it was 23/444 (5.2%). There was no statistical heterogeneity among the studies (P = 0.63, I2 = 2%). A fixed-effects model was used to combine the effect sizes for the Meta-analysis, which indicated that there was no statistically significant difference in the overall complication rate between the RTAPP group and the LTAPP group [OR = 1.22, 95% CI (0.68, 2.18), P = 0.51], as shown in Figure 6.

Readmission rate

Five studies [12-14,18,21] reported the readmission rate. The readmission rate in the RTAPP group was 12/672 (1.8%), while in the LTAPP group, it was 11/546 (2.0%). There was statistical heterogeneity among the studies (P = 0.06, I2 = 64%). A random-effects model was used to combine the effect sizes for the Meta-analysis, which indicated that there was no statistically significant difference in the readmission rate between the RTAPP group and the LTAPP group [OR = 1.31, 95% CI (0.23, 7.47), P = 0.76]. Sensitivity analysis, performed by sequentially excluding individual included studies, demonstrated that the direction of the combined effect value remained unchanged after each exclusion, suggesting the overall stability of the study results, as shown

in Figure 7.

Recurrence rate

Five studies [12,13,16,19,20] reported the recurrence rate. The recurrence rate in the RTAPP group was 3/359 (0.8%), while in the LTAPP group, it was 4/405 (0.9%). There was no statistical heterogeneity among the studies (P = 0.75, I2 = 0%). A fixed-effects model was used to combine the effect sizes for the Meta-analysis, which indicated that there was no statistically significant difference in the recurrence rate between the RTAPP group and the LTAPP group [OR = 0.82, 95% CI (0.22, 3.07), P = 0.77] , as shown in Figure 8.

Discussion

Inguinal hernia is a common condition in general surgery, with approximately 20 million inguinal hernia repair surgeries performed annually [26]. Traditionally, open and laparoscopic techniques have been the primary methods for inguinal hernia repair [27]. Laparoscopic hernia repair has gained significant attention from surgeons and patient preference due to its advantages, including reduced trauma, lower infection rates, and shorter postoperative recovery times [28]. The introduction of robotic surgical systems has greatly advanced the field of minimally invasive surgery [29]. As robotic technology continues to improve and new surgical instruments are developed, along with surgeons becoming more familiar with the system, its application has gradually expanded to inguinal hernia repair [30]. Currently, research on the comparison between robotic and laparoscopic inguinal hernia repair procedures is limited in sample size, and thus, the feasibility, safety, effectiveness, and cost-effectiveness of robotics in inguinal hernia repair have not been fully determined [12][31]. Therefore, this study aims to conduct a meta-analysis on the feasibility, safety, effectiveness, and cost-effectiveness of robotic and laparoscopic transabdominal preperitoneal repair in inguinal hernia repair.

This meta-analysis included a total of 10 studies, comprising 2 randomized controlled studies and 8 non-randomized controlled studies. From the findings of this meta-analysis, it is evident that RTAPP has a significantly longer operation time for inguinal hernia compared to LTAPP. The underlying reasons for this discrepancy may be attributed to two key factors: firstly, the introduction of robotic surgery occurred relatively later, and many of the studies included in this analysis represent early experiences with this technology; secondly, the robotic operating system necessitates a certain amount of preparation time [32]. As surgeons, and even the entire surgical team, become increasingly familiar with the robotic instruments and procedures in the operating room, and as the volume of surgical cases increases, it is possible that the operation time may decrease. In this meta-analysis, three studies reported on cost, with the research conducted in the United States and South Korea. It was found that the cost of robotic surgery was higher than that of laparoscopic surgery, and this difference was statistically significant. While the medical expenses associated with traditional tension-free inguinal hernia repair and laparoscopic hernia repair have gained wide

acceptance among patients, the high cost of robotic surgery represents a notable drawback. The primary factors contributing to the cost difference are the expenses associated with medical equipment and the longer operation time [31]. This has, to a certain extent, limited the application of this technology to patients in underdeveloped regions and those without health insurance coverage. However, in the future, as healthcare systems become more comprehensive and operation time decrease, cost may no longer be a significant concern. The anatomical structures in the inguinal region are complex, with numerous blood vessels and nerves [33]. Robotic surgical systems utilize their three-dimensional high-definition imaging systems, providing a clear surgical field and enhancing the surgeon's visual perception, similar to what is experienced in open surgery. This improvement addresses the limitations of laparoscopic surgery, which can only offer two-dimensional images. Robotic surgery ensures stable and precise surgical maneuvers [34], allowing for the protection of critical anatomical structures in the inguinal region. It significantly reduces the risk of intraoperative damage to structures such as the spermatic vessels, vas deferens, femoral vessels, and nerves. This meta-analysis suggests that there is no statistically significant difference between robotic and laparoscopic approaches in terms of overall complication rate, incidence of seroma, and readmission rate. Additionally, it does not lead to an increase in hospital stay, indicating the safety of RTAPP for inguinal hernia. The success of surgery also takes into account the recurrence rate, which is influenced by factors such as the surgeon's experience, mesh size, sufficient overlap of the hernia defect area, surgical site infection, and misdiagnosed hernias [35-37]. In this meta-analysis, the recurrence rate in the RTAPP group was 0.8%, while the LTAPP group had a recurrence rate of 0.9%. There was no statistically significant difference in recurrence rates between the two groups, indicating that RTAPP for inguinal hernia repair does not increase the postoperative recurrence risk and is effective. However, it should be noted that some studies had relatively short follow-up periods, and further research with longer follow-up times is needed to assess recurrence rates more comprehensively in the future.

The strength of evidence in the results of this meta-analysis may be influenced by the following factors: (1) A limited number of included studies with relatively small sample sizes. (2) Inclusion of only English-language literature, potentially leading to language bias. (3) There is significant heterogeneity among the included studies in terms of operation time, hospital stay, cost, and readmission rate. This heterogeneity is likely attributed to differences in surgeon expertise, surgical procedures, mesh materials, and fixation methods among the included studies, which inevitably impact the outcomes. (4) Inconsistency in follow-up durations across studies, with short-term follow-up being insufficient to assess and compare hernia recurrence between the two groups. (5) Some studies may not have adequately emphasized the importance of randomization, blinding, and allocation concealment in their randomized controlled trials, which, to a certain extent, could affect the strength of evidence in this study.

Comparing RTAPP inguinal hernia repair to LTAPP inguinal hernia repair, it appears to be a safe and viable alternative for the treatment of inguinal hernia, providing a new option. However, it is associated with a longer operation time and higher cost. Due to the limitations of this study, our conclusions still need validation through large-sample, multicenter, rigorously designed, high-quality clinical RCTs.

Acknowledgements

None.

Funding

No funding was received for this study.

Conflicts of interest statement

The authors have no conflicts of interest to declare that are relevant to the content of this article.

Ethics approval and consent to participate

An ethics statement is not applicable because this study is based exclusively on published literature.

Data availability statement

Data can be obtained from the original articles included in this meta-analysis.

Author Contributions

Conceptualization: Hui Dong, Li Li, Deng-Chao Wang, Jian Wei.

Data curation: Hui Dong, Li Li, Yue-Juan Li.

Formal analysis: Hui Dong, Li Li, Yue-Juan Li.

Investigation: Hui Dong, Li Li.

Methodology: Hui Dong, Li Li, Yue-Juan Li, Deng-Chao Wang, Jian Wei.

Project administration: Hui Dong, Jian Wei.

Resources: Hui Dong, Li Li.

Software: Hui Dong, Li Li, Yue-Juan Li, Deng-Chao Wang.

Supervision: Hui Dong, Jian Wei.

Validation: Hui Dong, Li Li, Yue-Juan Li, Deng-Chao Wang.

Visualization: Hui Dong, Li Li, Yue-Juan Li, Deng-Chao Wang.

Writing – original draft: Hui Dong, Li Li.

Writing – review & editing: Hui Dong, Deng-Chao Wang, Jian Wei.

References

1. Jensen KK, Henriksen NA, Jorgensen LN. Inguinal hernia epidemiology. Textbook of hernia. 2017: 23-27.
2. Patel V, Gupta T, El-Medani F, Gupta S. Laparoscopic inguinal hernia repair: transabdominal preperitoneal or totally extraperitoneal? Results of a 14-year prospective study. Chirurgia (Bucur). 2020, 115(5): 600-608.
3. Wang G, Zeng Y, Sheng X. Personnel Management in the Robotic Surgery Room. Robotic Surgery and Nursing. 2021: 111-115.
4. Comparetto C, Borruto F. Applications of Robotics in Gynecological Surgery//Design and Control Advances in Robotics. IGI Global. 2023: 256-294.
5. Iraniha A, Peloquin J. Long-term quality of life and outcomes following robotic assisted TAPP inguinal hernia repair. Journal of robotic surgery. 2018, 12(2): 261-269.
6. Aiolfi A, Cavalli M, Micheletto G, Bruni PG, Lombardo F, Perali C, et al. Robotic inguinal hernia repair: is technology taking over? Systematic review and meta-analysis. Hernia. 2019, 23: 509-519.
7. Hagen M E, Stein H, Curet M J. Introduction to the robotic system. Robotics in general surgery. 2014: 9-15.
8. Umari P, Mazzone E, De Groote R, Maes K, Mottrie A. Robot-Assisted Surgery. Urologic Surgery in the Digital Era: Next Generation Surgery and Novel Pathways. 2021: 129-158.
9. Costa TN, Tustumi F, Ferros LSM, Colonno BB, Abdalla RZ, , et al. Robotic-assisted versus laparoscopic incisional hernia repair: differences in direct costs from a Brazilian public institute perspective. ABCD. Arquivos Brasileiros de Cirurgia Digestiva (São Paulo). 2023, 35.
10. Vasudevan V, Reusche R, Nelson E, Kaza S. Robotic paraesophageal hernia repair: a single-center experience and systematic review. Journal of Robotic Surgery. 2018, 12: 81-86.
11. Podolsky D, Novitsky Y. Robotic inguinal hernia repair. Surgical Clinics. 2020, 100(2): 409-415.
12. Ayuso SA, Marturano MN, Katzen MM, Aladegbami BG, Augenstein VA. Laparoscopic versus robotic inguinal hernia repair: a single-center case-matched study. Surgical Endoscopy. 2023, 37(1): 631-637.
13. Choi YS, Kim KD, Choi MS, Heo YS, Yi JW, Choe YM. Initial Experience of Robot-Assisted Transabdominal Preperitoneal (TAPP) Inguinal Hernia Repair by a Single Surgeon in South Korea. Medicina. 2023, 59(3): 582.
14. Gerdes S, Burger R, Liesch G, Freitag B, Serra M, Vonlanthen R, et al. Results of robotic TAPP and conventional laparoscopic TAPP in an outpatient setting: a cohort study in Switzerland. Langenbeck's Archives of Surgery. 2022, 407(6): 2563-2567.
15. Hsu JL, Kelley JK, Zambito GM, Korakavi N, Phillips A, Phillips M, et al. Comparison of Robotic, Laparoscopic, and Open Unilateral Repair of Non-recurrent Inguinal Hernia. The American Surgeon. 2022: 00031348221136572.

16. Miller BT, Prabhu AS, Petro CC, Beffa LRA, Carbonell AM, Hope W, et al. Laparoscopic versus robotic inguinal hernia repair: 1-and 2-year outcomes from the RIVAL trial. Surgical endoscopy. 2023, 37(1): 723-728.
17. Muysoms F, Van Cleven S, Kyle-Leinhase I, Ballecer C, Ramaswamy A. Robotic-assisted laparoscopic groin hernia repair: observational case-control study on the operative time during the learning curve. Surgical Endoscopy. 2018, 32: 4850-4859.
18. Muysoms F, Vierstraete M, Nachtergaele F, Van Garsse S, Pletinckx P, Ramaswamy A. Economic assessment of starting robot-assisted laparoscopic inguinal hernia repair in a single-centre retrospective comparative study: the EASTER study. BJS open. 2021, 5(1): zraa046.
19. Okamoto N, Mineta S, Mishima K, Fujiyama Y, Wakabayashi T, Fujita S, et al. Comparison of short-term outcomes of robotic and laparoscopic transabdominal peritoneal repair for unilateral inguinal hernia: a propensity-score matched analysis. Hernia. 2023: 1-8.
20. Peltrini R, Corcione F, Pacella D, Castiglioni S, Lionetti R, Andreuccetti J, et al. Robotic versus laparoscopic transabdominal preperitoneal (TAPP) approaches to bilateral hernia repair: a multicenter retrospective study using propensity score matching analysis. Surgical Endoscopy. 2023, 37(2): 1188-1193.
21. Prabhu AS, Carbonell A, Hope W, Warren J, Higgins R, Jacob B, et al. Robotic inguinal vs transabdominal laparoscopic inguinal hernia repair: the RIVAL randomized clinical trial. JAMA surgery. 2020, 155(5): 380-387.
22. Page MJ, McKenzie JE, Bossuyt PM, Boutron I, Hoffmann TC, Mulrow CD, et al. The PRISMA 2020 statement: an updated guideline for reporting systematic reviews. Int J Surg. 2021, 88: 105906.
23. Jadad AR, Moore RA, Carroll D, Jenkinson C, Reynolds DJM ,Gavaghan DJ, et al. Assessing the quality of reports of randomized clinical trial:is blinding necessary?Control Clin Trials. 1996, 17 (1) :1-12.
24. Stang A. Critical evaluation of the Newcastle-Ottawa scale for the assessment of the quality of nonrandomized studies in meta-analyses.Eur J Epidemiol. 2010, 25(9) : 603-605.
25. Sedgwick P, Marston L. How to read a funnel plot in a meta- analysis. BMJ. 2015:351.
26. Merola G, Cavallaro G, Iorio O, Frascio M, Pontecorvi E, Corcione F, et al. Learning curve in open inguinal hernia repair: a quality improvement multicentre study about Lichtenstein technique. Hernia. 2020, 24: 651-659.
27. Haladu N, Alabi A, Brazzelli M, Imamura M, Ahmed I, Ramsay G, et al. Open versus laparoscopic repair of inguinal hernia: an overview of systematic reviews of randomised controlled trials. Surgical Endoscopy. 2022, 36(7): 4685-4700.
28. Pang NQ, Ng CSY, Wong CJH. Laparoscopic versus open groin hernia repair in older adults: a systematic review and meta- analysis. ANZ Journal of Surgery. 2022, 92(10): 2457-2463.
29. Perez RE, Schwaitzberg SD. Robotic surgery: finding value in 2019 and beyond. Ann Laparosc Endosc Surg. 2019, 4(3): 1-7.

30. Ito F, Jarrard D, Gould JC. Transabdominal preperitoneal robotic inguinal hernia repair. Journal of laparoendoscopic & advanced surgical techniques. 2008, 18(3): 397-399.

31. Abdelmoaty WF, Dunst CM, Neighorn C, Swanstrom LL, Hammill CW. Robotic-assisted versus laparoscopic unilateral inguinal hernia repair: a comprehensive cost analysis. Surgical endoscopy. 2019, 33: 3436-3443.

32. Proietti F, La Regina D, Pini R, Giuseppe MD, Cianfarani A, Mongelli F. Learning curve of robotic-assisted transabdominal preperitoneal repair (rTAPP) for inguinal hernias. Surgical Endoscopy. 2021, 35: 6643-6649.

33. Amato G. Gross Anatomy of the Inguinal Region//Inguinal Hernia: Pathophysiology and Genesis of the Disease. Cham: Springer International Publishing. 2022: 1-14.

34. Morrell ALG, Morrell Junior AC, MENDES J, FREITAS M, MORRELL AG, MORRELL A. Robotic TAPP inguinal hernia repair: lessons learned from 97 cases. Revista do Colégio Brasileiro de Cirurgiões. 2021, 48.

35. Burcharth J. The epidemiology and risk factors for recurrence after inguinal hernia surgery. Dan Med J. 2014, 61(5): B4846.

36. Siddaiah-Subramanya M, Ashrafi D, Memon B, Memon MA. Causes of recurrence in laparoscopic inguinal hernia repair. Hernia. 2018, 22(6): 975-986.

37. Niebuhr H, Köckerling F. Surgical risk factors for recurrence in inguinal hernia repair–a review of the literature. Innovative surgical sciences. 2017, 2(2): 53-59.

Figure legends

Fig. 1 Study selection.

Fig. 2 Forest plot comparing the operation time between the two groups.

Fig. 3 Forest plot comparing the hospital stay between the two groups.

Fig. 4 Forest plot comparing the cost between the two groups.

Fig. 5 Forest plot comparing the incidence of seroma between the two groups.

Fig. 6 Forest plot comparing the overall complication rate between the two groups.

Fig. 7 Forest plot comparing the readmission rate between the two groups.

Fig. 8 Forest plot comparing the recurrence rate between the two groups.

Fig. 1 Study selection.

Click here to access/download;Figure;Fig. 1.docx

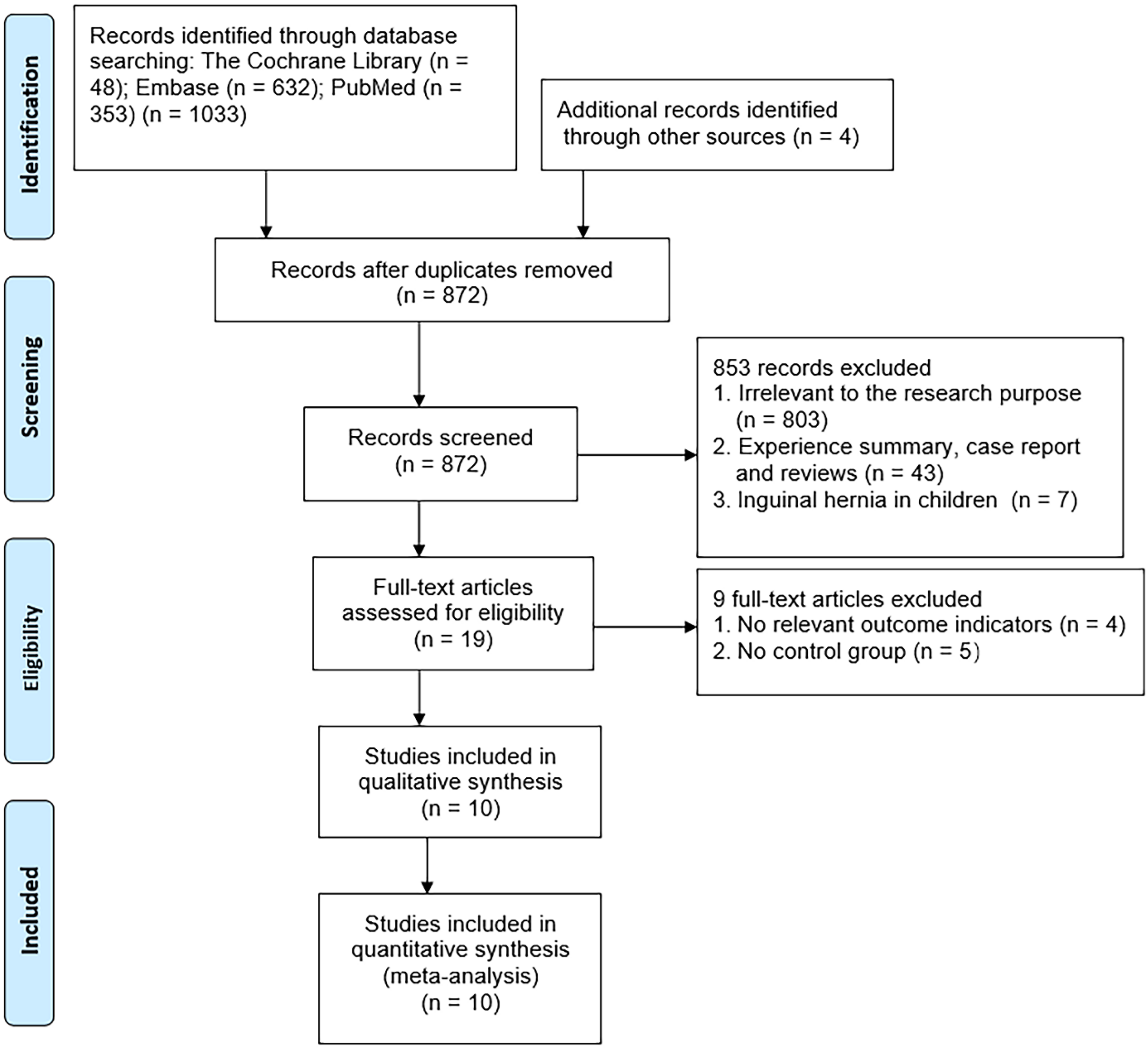

Fig. 1

Fig. 2 Forest plot comparing the operation time between the two groups.

Click here to access/download;Figure;Fig. 2.docx

	RTAPP			LTAPP				Mean Difference
**Study or Subgroup**	**Mean**	**SD**	**Total**	**Mean**	**SD**	**Total**	**Weight**	**IV, Random, 95% CI**
Ayuso 2023	109.1	50	141	100.6	38.3	141	13.6%	8.50 [-1.90, 18.90]
Choi 2023	30.2	11.9	50	31.5	10.3	50	17.5%	-1.30 [-5.66, 3.06]
Hsu 2022	62	4.3	207	50	4.8	212	18.6%	12.00 [11.13, 12.87]
Okamoto 2023	99.5	6.2	80	89	5.8	80	18.5%	10.50 [8.64, 12.36]
Peltrini 2023	98	29	40	79	21	80	13.8%	19.00 [8.90, 29.10]
Prabhu 2020	75.5	8.7	48	40.5	8.65	54	18.0%	35.00 [31.63, 38.37]
**Total (95% CI)**			**566**			**617**	**100.0%**	**14.02 [6.65, 21.39]**

Heterogeneity: Tau² = 75.73; Chi² = 217.19, df = 5 (P < 0.00001); I² = 98%
Test for overall effect: Z = 3.73 (P = 0.0002)

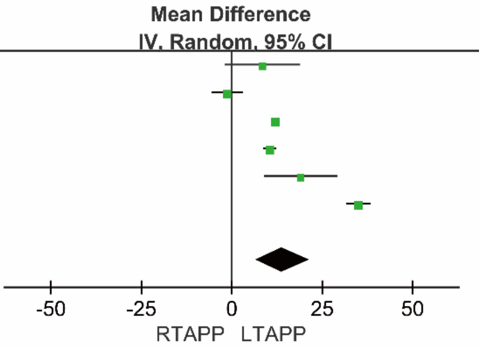

Fig. 2

Fig. 3 Forest plot comparing the hospital stay between the two groups.

Click here to access/download;Figure;Fig. 3.docx

	RTAPP			LTAPP				Mean Difference
Study or Subgroup	Mean	SD	Total	Mean	SD	Total	Weight	IV, Random, 95% CI
Ayuso 2023	1.3	1.1	141	1.2	0.5	141	43.6%	0.10 [-0.10, 0.30]
Choi 2023	3.4	0.6	50	3.5	2.2	50	15.8%	-0.10 [-0.73, 0.53]
Peltrini 2023	1.5	0.74	40	1.05	0.22	80	40.6%	0.45 [0.22, 0.68]
**Total (95% CI)**			**231**			**271**	**100.0%**	**0.21 [-0.09, 0.51]**

Heterogeneity: Tau² = 0.04; Chi² = 6.06, df = 2 (P = 0.05); I² = 67%

Test for overall effect: Z = 1.38 (P = 0.17)

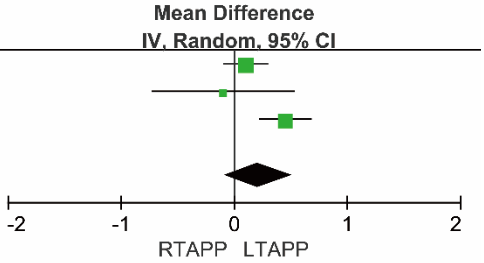

Fig. 3

Fig. 4 Forest plot comparing the cost between the two groups.

Click here to access/download;Figure;Fig. 4.docx

	RTAPP			LTAPP				Mean Difference
**Study or Subgroup**	**Mean**	**SD**	**Total**	**Mean**	**SD**	**Total**	**Weight**	**IV, Random, 95% CI**
Ayuso 2023	52.07	13.77	141	41.55	8.85	141	18.6%	10.52 [7.82, 13.22]
Choi 2023	3.81	0.17	50	0.21	0.03	50	40.7%	3.60 [3.55, 3.65]
Prabhu 2020	3.26	0.39	48	1.42	0.18	54	40.6%	1.84 [1.72, 1.96]
**Total (95% CI)**			**239**			**245**	**100.0%**	**4.17 [2.59, 5.76]**

Heterogeneity: $Tau^2 = 1.60$; $Chi^2 = 736.66$, $df = 2$ ($P < 0.00001$); $I^2 = 100\%$

Test for overall effect: $Z = 5.17$ ($P < 0.00001$)

Mean Difference
IV, Random, 95% CI

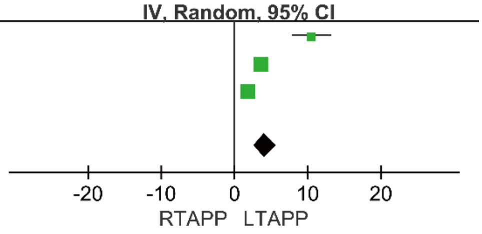

Fig. 4

Fig. 5 Forest plot comparing the incidence of seroma between the two groups.

Click here to access/download;Figure;Fig. 5.docx

	RTAPP		LTAPP			Odds Ratio
Study or Subgroup	Events	Total	Events	Total	Weight	M-H, Fixed, 95% CI
Choi 2023	2	50	0	50	2.2%	5.21 [0.24, 111.24]
Muysoms 2018	7	49	7	63	24.5%	1.33 [0.43, 4.09]
Okamoto 2023	9	80	14	80	58.1%	0.60 [0.24, 1.47]
Peltrini 2023	1	40	5	80	15.2%	0.38 [0.04, 3.41]
**Total (95% CI)**		**219**		**273**	**100.0%**	**0.85 [0.45, 1.59]**
Total events	19		26			

Heterogeneity: Chi² = 3.06, df = 3 (P = 0.38); I² = 2%

Test for overall effect: Z = 0.52 (P = 0.61)

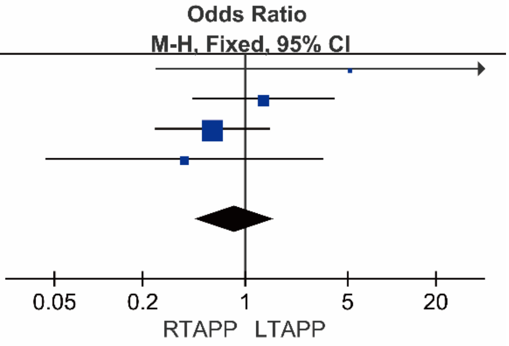

Fig. 5

Fig. 6 Forest plot comparing the overall complication rate between the two groups.

Click here to access/download;Figure;Fig. 6.docx

Study or Subgroup	RTAPP Events	RTAPP Total	LTAPP Events	LTAPP Total	Weight	Odds Ratio M-H, Fixed, 95% CI
Gerdes 2022	0	29	0	29		Not estimable
Muysoms 2018	5	49	3	63	11.4%	2.27 [0.52, 10.02]
Muysoms 2021	18	404	12	272	66.1%	1.01 [0.48, 2.13]
Peltrini 2023	5	40	8	80	22.5%	1.29 [0.39, 4.22]
**Total (95% CI)**		**522**		**444**	**100.0%**	**1.22 [0.68, 2.18]**
Total events	28		23			

Heterogeneity: Chi² = 0.93, df = 2 (P = 0.63); I² = 0%
Test for overall effect: Z = 0.66 (P = 0.51)

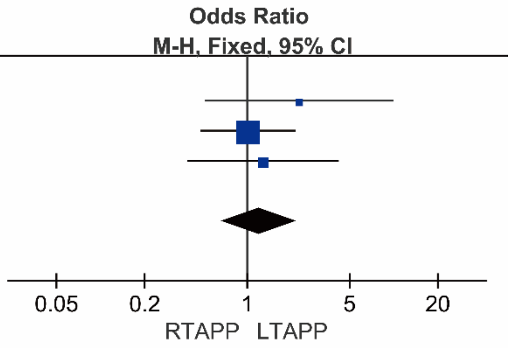

Fig. 6

Fig. 7 Forest plot comparing the readmission rate between the two groups.

Click here to access/download;Figure;Fig. 7.docx

Study or Subgroup	RTAPP Events	RTAPP Total	LTAPP Events	LTAPP Total	Weight	Odds Ratio M-H, Random, 95% CI
Ayuso 2023	3	141	0	141	20.9%	7.15 [0.37, 139.74]
Choi 2023	0	50	0	50		Not estimable
Gerdes 2022	0	29	0	29		Not estimable
Muysoms 2021	5	404	9	272	44.2%	0.37 [0.12, 1.10]
Prabhu 2020	4	48	2	54	34.9%	2.36 [0.41, 13.52]
**Total (95% CI)**		**672**		**546**	**100.0%**	**1.31 [0.23, 7.47]**
Total events	12		11			

Heterogeneity: Tau² = 1.47; Chi² = 5.58, df = 2 (P = 0.06); I² = 64%

Test for overall effect: Z = 0.30 (P = 0.76)

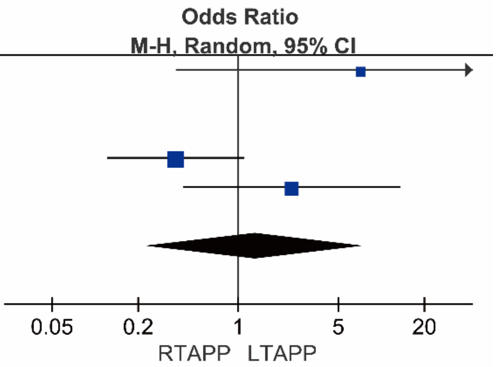

Fig. 7

Fig. 8 Forest plot comparing the recurrence rate between the two groups.

Click here to access/download;Figure;Fig. 8.docx

	RTAPP		LTAPP			Odds Ratio
Study or Subgroup	Events	Total	Events	Total	Weight	M-H, Fixed, 95% CI
Ayuso 2023	2	141	1	141	20.2%	2.01 [0.18, 22.47]
Choi 2023	0	50	1	50	30.4%	0.33 [0.01, 8.21]
Miller 2023	1	48	1	54	18.9%	1.13 [0.07, 18.53]
Okamoto 2023	0	80	1	80	30.5%	0.33 [0.01, 8.20]
Peltrini 2023	0	40	0	80		Not estimable
**Total (95% CI)**		**359**		**405**	**100.0%**	**0.82 [0.22, 3.07]**
Total events	3		4			

Heterogeneity: Chi² = 1.21, df = 3 (P = 0.75); I² = 0%
Test for overall effect: Z = 0.30 (P = 0.77)

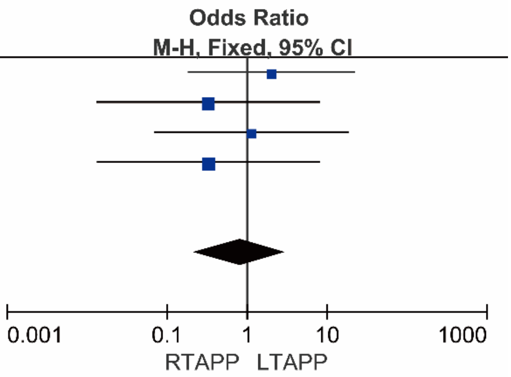

Fig. 8

Table 1. Characteristics of the studies included in this meta-analysis

Click here to access/download
Supporting Information
Table 1.docx

PRISMA checklist

Click here to access/download
Supporting Information
PRISMA_2020_checklist.docx
